# Recent advances of m6A methylation in skeletal system disease

**DOI:** 10.1186/s12967-024-04944-y

**Published:** 2024-02-14

**Authors:** Jianhui Liang, Qian Yi, Yang Liu, Jiachen Li, Zecheng Yang, Wei Sun, Weichao Sun

**Affiliations:** 1 Department of Orthopedics, Shenzhen Second People’s Hospital/First Affiliated Hospital of Shenzhen University Health Science Center, Shenzhen, 518035 Guangdong China; 2 Shantou University Medical College, Shantou, 515000 China; 3 Department of Physiology, School of Basic Medical Science, Southwest Medical University, Luzhou, 646099 Sichuan China; 4 The Central Laboratory, Shenzhen Second People’s Hospital/First Affiliated Hospital of Shenzhen University Health Science Center, Shenzhen, 518035 Guangdong China

**Keywords:** m6A modification, Osteosarcoma, Osteoporosis, Rheumatoid arthritis, Osteoarthritis

## Abstract

Skeletal system disease (SSD) is defined as a class of chronic disorders of skeletal system with poor prognosis and causes heavy economic burden. m6A, methylation at the N6 position of adenosine in RNA, is a reversible and dynamic modification in posttranscriptional mRNA. Evidences suggest that m6A modifications play a crucial role in regulating biological processes of all kinds of diseases, such as malignancy. Recently studies have revealed that as the most abundant epigentic modification, m6A is involved in the progression of SSD. However, the function of m6A modification in SSD is not fully illustrated. Therefore, make clear the relationship between m6A modification and SSD pathogenesis might provide novel sights for prevention and targeted treatment of SSD. This article will summarize the recent advances of m6A regulation in the biological processes of SSD, including osteoporosis, osteosarcoma, rheumatoid arthritis and osteoarthritis, and discuss the potential clinical value, research challenge and future prospect of m6A modification in SSD.

## Introduction

Skeletal system disease (SSD) is defined as a class of chronic disorders of skeletal system, such as bone, cartilage and joint, characterized by cartilage destruction, limitation of movement and significant disability, and commonly includes osteoporosis (OP), osteosarcoma (OS), rheumatoid arthritis (RA), osteoarthritis (OA) and so on [1–4]. As the incidence of SSD is increasing year by year, it has become one of the heaviest burdens on global health and economics [5]. In recent decades, SSD has gained increasing attention, however, the pathogenesis remains unsystematically discussed and few effective therapeutic options for patients [6]. Therefore, it is imperative to understand the molecular mechanism of SSD and more efforts are needed to develop new therapeutic strategies.

N6‐methyladenosine (m6A) modification, involved in posttranscriptional RNA regulation, is the most common epigenetic modification that perpetuate alternative gene expression and function without changing gene sequence [7, 8]. And it is widely involved in regulating RNA metabolism, such as RNA splicing, transportation, localization, translation, degradation and so on [9, 10]. As the most abundant modification of mRNA and lncRNA among mammals, m6A modification plays a critical role in understanding the pathogenesis of diseases [11, 12]. For example, it has been found that m6A modification can regulate glioblastoma stem cells growth and self-renewal [13]. Therefore, m6A modification has become a popular research topic and researchers have made great progress in exploring pathogenesis and new therapeutic strategies. Similarly, current studies have found m6A modification plays a key role in the occurrence and development of SSD [14]. Such as Pan et al. found that silencing of WTAP retarded OS progress which could be partially eliminated by knockdown of ALB [15].

Therefore, in the current work, we summarize the latest research concerning roles that m6A plays in common SSD including osteoporosis, osteosarcoma, rheumatoid arthritis and osteoarthritis. And we also discuss the novel potential of m6A modification as a therapeutic target of SSD.

## Enzymes and proteins involved in modification by m6A

**Fig. 1:**
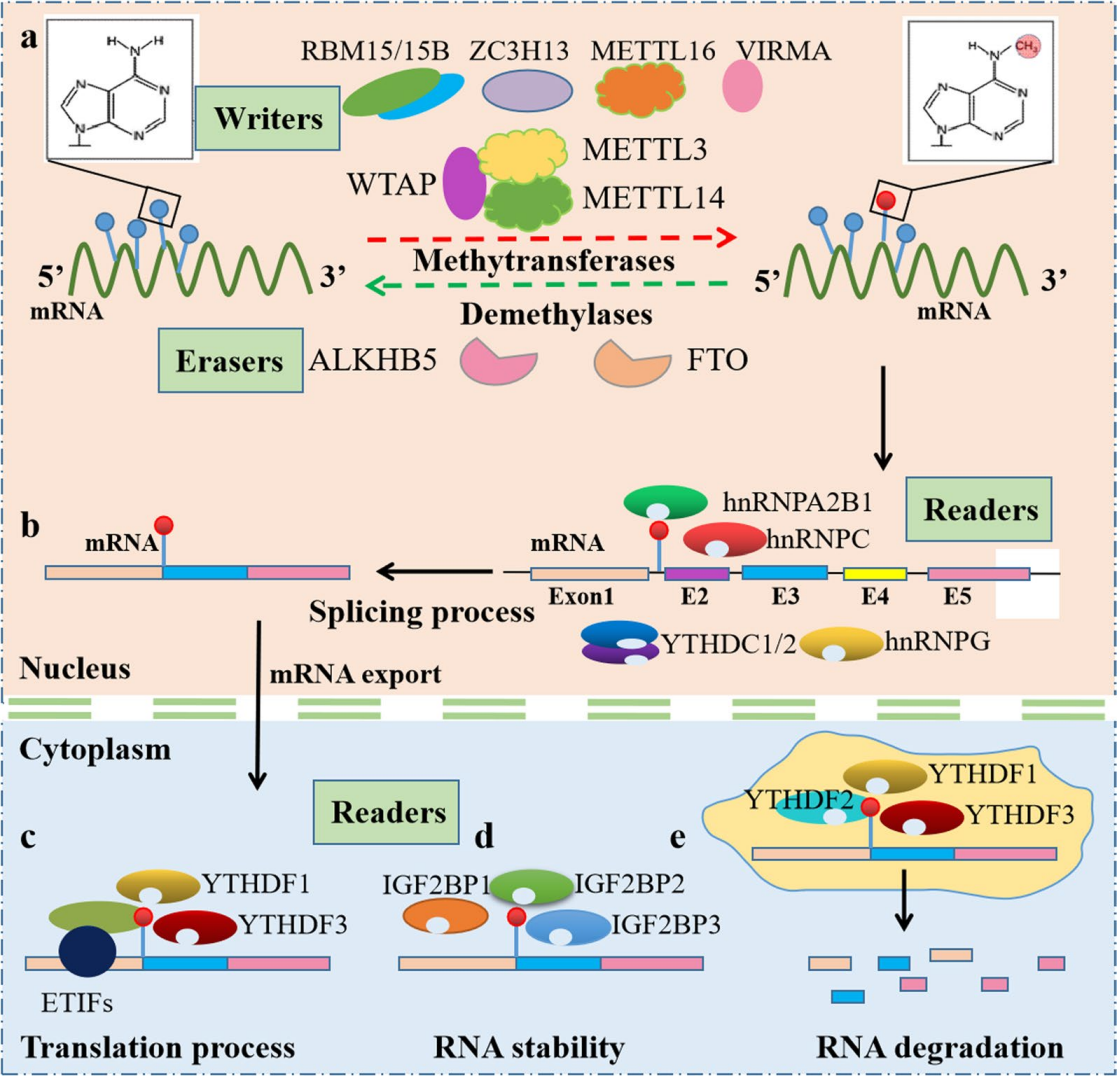
Structural schematic diagram of reversible and dynamic posttranscriptional m6A modification of RNA. **a**. In the nucleus, methyltransferases and demethylases regulated the m6A modifications of target mRNAs. **b**. In the nucleus, m6A readers such as hnRNPA2B1 regulated the splicing process of target mRNAs. **c**. In the cytoplasm, m6A readers such as YTHDF1 regulated the translation process of target mRNAs. **d**. In the cytoplasm, m6A readers such as IGF2BP1 regulated the stability of target mRNAs. **e**. In the cytoplasm, m6A readers such as YTHDF3 mediated the degradation of target mRNAs

m6A methylation, first discovered in 1974 [16], can be understood simply that a methyl group is added to the sixth position of the nitrogen atom of adenosine on RNA [17]. It is shown that m6A methylation is a reversible and dynamic posttranscriptional modification of RNA which is distinct from other kinds of epigenetic modifications [18] (Fig. 1). There are three kinds of enzymes and proteins involved in m6A modification, including writers, erasers and, readers [19]. Firstly, m6A writers, means methyltransferases, include methyltransferase-like 3 (METTL3), METTL5, METTL14, METTL16, Wilm’s tumor 1-associated protein (WTAP), RBM15/15B, ZC3H13 and VIRMA, also called KIAA1429. Secondly, m6A erasers, means demethylases, include fat mass and obesity-associated protein (FTO) and α-ketoglutarate-dependent dioxygenase alk B homolog 5 (ALKBH5). Lastly, m6A readers are kind of RNA binding proteins (RBPs) which could recognize and bind to the m6A modification position of RNA. These RBPs consisted of YT521-B homology (YTH) domain family (YTHDF1/2/3, YTHDC1/2), heterogeneous nuclear ribonucleoproteins (hnRNP) family (HNRNPC/G/A2B1), insulin-like growth factor 2 mRNA-binding proteins family (IGF2BP1/2/3) and ELAV-like protein 1 (ELAVL1) [20–23].

### m6A writers

METTL3, first identified m6A methyltransferase [24], is the only catalytic subunit of the methyltransferase complex (MTC) by which writers promote m6A methylation on posttranscriptional RNA [25]. METTL14, the most important accessory subunit of MTC, can enhance the catalytic activity of METTL3 and stabilize MTC [26]. Moreover, METTL3 has no catalytic activity without the help of METTL14 and stable METTL3-METTL14 complex is the core component of MTC [27, 28]. WTAP, another accessory subunit, has been proved to regulate m6A methylation by recognizing and recruiting METTL3-METTL14 complex to target RNA [29]. RBM15/15B, RNA binding domain protein, can work with WTAP to recruit m6A complex to RNA binding sites [30]. Besides, it has been found that METTL5 and METTL16 catalyze m6A methylation independent of the MTC, METTL5 is involved in rRNA m6A [31] and METTL16 promotes depositing m6A, splicing and translation of mRNA transcripts [32]. Moreover, ZC3H13 is responsible for m6A installation and plays critical roles in the proliferation of cancer cells [33]. VIRMA, also called KIAA1429, is important for specially depositing m6A methylation to 3′UTR region of mRNA [34, 35].

### m6A erasers

m6A erasers, demethylases, remove m6A modifications from target RNA and are essential to mediate the reversible and dynamic process of m6A methylation [36, 37]. FTO is the first m6A eraser which was discovered in 2011 [38]. The demethylation mechanism of FTO is to firstly oxidate m6A to N6-hydroxymethyladenosine (hm6A), secondly transform hm6A into N6-formyladenosine (f6A), finally change f6A into adenosine [39]. It is proved that FTO downregulation cooperating with YTHDF2 promotes mRNA degradation of ATG5 and ATG7 [40] and FTO enhances STAT3 mRNA stability in a demethylation-mediated m6A manner [41]. Luo et al. reported that FTO regulated ADRB1 mRNA level through mRNA alternative splicing [42]. As the second demethylase discovered in 2013 [43], ALKBH5 can catalyze m6A into adenosine directly which is different from FTO [44]. Studies have shown that ALKBH5 upregulated, HuR bound to the unmethylated 3′UTR and then promoted the expression of FOXM1 nascent transcripts [45], meanwhile, knockout of ALKBH5 inhibited he translation efficiency of FOXM1 mRNA [46]. Moreover, ALKBH5 affects mRNA stability rather than translation to regulate its targets’ expression level [47].

### m6A readers

m6A readers, effectively binding to and recognizing m6A, make sure that m6A can regulate the metabolism of m6A-modified RNA [48]. YTH domain family is the most common “reader” of m6A and YTHDF2 is the first identified reader [49]. YTHDF1-3 are highly homologous in the sequence of their YTH domain but play different roles in processing mRNA [37]. YTHDF1 promotes mRNA translation whereas YTHDF2 catalyzes the degradation of mRNA, and YTHDF3 participates in the metabolism of mRNA in synergy with YTHDF1 and YTHDF2 [50]. For example, Bai et al. found that YTHDF2 promotes its degradation in an m6A-dependent manner through binds to the 3'-UTR of DAPK3 mRNA [51]. It is reported that YTHDF3 and YTHDF1 interactions with proteins associated with mRNA translation are blocked by O-GlcNAcylation, which inhibits the translation-promoting function [52]. Furthermore, YTHDC1-2 were another important m6A reader involved in RNA-related processes [53, 54]. For example, YTHDC1 works as a splicing protein alternatively splicing its targets’ mRNA [55]. And YTHDC2 was reported to recognize and bind to the m6A site "GGACA" in LIMK1 mRNA, thereby increasing LIMK1 mRNA stability and expression [56].

In addition to YTH domain family, there are other m6A readers involved in RNA splicing, translation and transport [57]. It has been reported that HNRNPC and HNRNPG regulate RNA metabolism via m6A switch, which is distinct from other m6A readers [58]. As for m6A switch, m6A-induced RNA structural alteration, was first identified in 2015 and can promote HNRNP proteins binding to mRNA for RNA processing [58, 59]. Conversely, HNRNPA2B1 binds to mRNA directly, not through m6A switch [60]. It is reported that HNRNPA2B1 binds to Specific RNA substrates and DNA motifs and then regulates RNA metabolism processes [61]. Moreover, IGF2BP1-3 cooperate with ELAVL1 to enhance the stability and translation efficiency of mRNA [62]. For example, it has been reported that IGF2BP1 coworking with ELAVL1 enhances the stability of MIR210HG [63]. IGF2BP3/ELAVL1 complex recognizes and enhances mRNA stability which prolongs half-lives of the mRNA molecules and increases target genes expression [64]. Furthermore, SR family is another kind of RNA binding proteins regulating RNA alternative splicing [65]. And it is reported that SRSF9 binds to and stabilizes DSN1 mRNA in an m6A-related manner, which is weakened by METTL3 downregulation [66].

## m6A and skeletal system disease

### m6A and osteoarthritis

OA, mainly characterized by progressive cartilage degeneration and synovial inflammation in pathology, is the most common chronic, degenerative joint disease in aging population and the symptom of OA is pain, stiffness, limitation of movement and joint deformity [67, 68]. The exact pathogenesis of OA is still ambiguous and the role of m6A modifications in OA has attracted great attention of researchers in recent 5 years. We show in Table 1 the function m6A regulation in osteoarthritis.

**Table 1:** Role of m6A modulators in osteoarthritis

m6A regulators	Cells	Animals	Expression	Targets	Biological function	Reference
WTAP	Human articular chondrocyte	DMM mouse model	Up	WTAP/YTHDF2/ miR-92b-5p/TIMP4	OA progression	[69]
METTL3	Human articular chondrocyte	–	Up	METTL3/NEK7	Chondrocytes pyroptosis	[70]
METTL3	Human articular chondrocyte	ACL-T mouse model	Up	METTL3/IGF2BP2/ STAT1/ADAMTS12	Cartilage damage	[71]
METTL3	Human primary chondrocyte	MIA mouse model	–	METTL3/IGFBP7-OT/ DNMT1/ DNMT3a-IGFBP7	Chondrocytes apoptosis	[72]
METTL3	Human articular chondrocyte	DMM mouse model	–	RPL38/METTL3/ SOCS2/JAK2/STAT3	Inflammatory response	[73]
METTL3	Human articular chondrocyte	DMM mouse model	–	miR-1208/METTL3/ NLRP3	Enhanced proliferation and migration, inhibited apoptosis	[74]
METTL3	Human articular chondrocyte	DMM mouse model	Down	CREB/miR-373/ METTL3/TFEB	Autophagy	[75]
METTL3	Human articular chondrocyte	–	Up	METTL3/LINC00680/SIRT1	OA progression	[76]
METTL3	Human endplate chondrocyte	Sprague Dawley rat	Up	METTL3/Sox9	OA progression	[77]
METTL3	Human fibroblast-like synoviocytes	DMM mouse model	–	METTL3/YTHDF2/ ATG7/GATA4	Autophagy	[78]
METTL3	ATDC5 cell	MIA mouse model	Down	METTL3/YTHDF1/ Bcl2	Inhibited the apoptosis and autophagy	[79]
FTO	Human articular chondrocyte	DMM mouse model	Down	FTO/miR-3591-5p/ PRKAA2	Alleviated cartilage damage	[80]
FTO	C28/I2 cell	MIA mouse model	Down	FTO/miR-515-5p/ TLR4/MyD88/NF-κB	Reduced apoptosis, inhibited inflammation	[81]
FTO	Mouse chondrocyte	Sprague Dawley rat	Up	OANCT /FTO/ PIK3R5/PI3K/AKT/mTOR pathway	OA progression	[82]
FTO	Human articular chondrocyte	MIA mouse model	Down	FTO/AC008/ miR-328-3p‒AQP1/ ANKH	OA progression	[83]
ALKBH5	Human articular chondrocyte	–	DOWN	ALKBH5/YTHDF2/ HS3ST3B1-IT1/ HS3ST3B1	OA progression	[84]
ALKBH5	Human bone marrow MSCs	ACLT mouse model	DOWN	ALKBH5/IGF2BP1/ CYP1B1 mRNA	MSC senescence	[85]
ALKBH5	Human articular chondrocyte	DMM mouse model	UP	ALKBH5/ MiR-654-3p/ TNFRSF9/ NF-κB	OA progression	[86]

Increasing evidences have shown that METTL3, WTAP, FTO and ALKBH5 are aberrantly expressed in OA chondrocytes and involved in OA pathogenesis by regulating chondrocyte proliferation, apoptosis, extracellular matrix (ECM) degradation through related signal pathways [69, 72, 80, 84]. For example, Lin et al. have revealed that WTAP-mediated miR-92b-5p/TIMP4 axis plays crucial role in OA development. Overexpression of WTAP suppressed cell proliferation enhanced apoptosis and ECM degradation in an LPS-induced OA chondrocyte model and promoting cartilage damage in a destabilizing the medial meniscus (DMM)-induced OA mice model [69]. Furthermore, the fibroblast-like synoviocyte (FLS) senescence is tightly associated with OA progression. It has been displayed that autophagy-related 7 (ATG7) mRNA regulates FLS senescence through autophagy-GATA4 axis in an METTL3/YTHDF2 dependent manner. And targeted METTL3 inhibition has been proved to enhance autophagy and reduce senescence- associated secretory phenotype expression in senescent FLS to decelerate OA development in DMM-induced mice model [78]. Furthermore, Lv et al. found that FTO cooperated with exosomal OANCT from dysfunctional chondrocytes could affect PIK3R5 mRNA stability, and then promoted OA progression via PI3K/AKT/mTOR pathway [82].

Lange-Brokaar’s study found that immunocytes, including T cells, B cells, NK cells and so on, contributed to cartilage injury and repair [87]. Recently study revealed that m6A was proved involved in OA progression by regulating immune responses [88]. Evidence found that IGFBP1 and RBM15B were strongly correlated with infiltrating immunocytes [89], YTHDF2 was positively related with regulatory T cells, IGFBP2 was negatively associated with dendritic cells [90], and IGF2BP3 upregulation promoted macrophage M1 polarization in OA [91].

Long non-coding RNA (lncRNA), transcript lacking protein-coding ability but mediating gene expression, is believed to play an essential role in OA development [92, 93]. It has been found that METTL3-mediated upregulation of IGFBP7-OT via DNMT1/DNMT3a-IGFBP7 axis promotes OA progression [72]. Moreover, Ren et al. found that METTL3 overexpression increased the LINC0068 level in OA, which repressed chondrocyte proliferation and accelerated ECM degradation [76]. Conversely, ALKBH5-mediated upregulation of HS3ST3B1-IT1 suppresses OA progression [84]. What’s more, Yang et al. showed that FTO suppressed the expression of AC008, which promoted chondrocyte apoptosis and ECM degradation in OA [83]. The above findings suggest that the relationship between m6A and lncRNA may provide new ideas for the future therapy of OA.

Recent studies mainly focus on the regulatory role of METTL3 in OA and the molecular mechanisms of METTL3 in OA progress are revealed in detail. Therefore, METTL3 may serve as potential therapeutic targets to alleviate OA [73]. For example, Xiong et al. proved that METTL3 interacted with NEK7 to inhibit OA progress [70]. Zhou et al. reported that hucMSCs-EVs could alleviate OA through combining with METTL3 to lower the m6A level of NLRP3 mRNA [74]. And BMSC-Exos were applied to prevent OA progression via disrupting METTL3-m6A-ACSL4 axis [94]. In addition, FTO and ALKBH5 are identified as potential targets to alleviate OA. For example, Liu et al. reported that overexpression of FTO inhibited the miR-3591-5p maturation via regulating demethylation of pri-miR-3591, and then downregulated PRKAA2 to alleviate cartilage damage in OA [80]. Cai and colleagues found that upregulating FTO promoted proliferation, inhibited apoptosis and inflammation in LPS-induced C28/I2 cells through the miR-515-5p/ TLR4/MyD88/NF-κB axis [81]. Moreover, senescent mesenchymal stem cell (MSC) can be another target to alleviate OA progression. Ye et al. have proved that overexpression of ALKBH5 inhibits MSC senescence by degrading CYP1B1 mRNA via IGF2BP1 [85].

However, there still some limitations in the study of m6A and osteoarthritis. Firstly, studies about m6A in regulation immune microenvironment of OA are mostly based on bioinformatics analysis [95, 96], they need to be verified by more experiments. Secondly, although many differentially expressed m6A regulators have been identified via bioinformatic analysis, only the mechanism of METTL3, WTAP, FTO, ALKBH5, YTHDF2 and IGF2BP3 in OA have been shown in recent studies. Therefore, more experiments are needed to reveal mechanisms of m6A regulators and their targets in OA. In addition, m6A regulators can be potential therapeutic targets for OA [97], but there are lack of studies exploring potential role of m6A regulators in diagnosis, treatment and prognosis of OA.

### m6A and rheumatoid arthritis

Rheumatoid arthritis (RA), characterized by persistent synovial inflammation and joint destruction, is the most common chronic autoimmune joint disorder and the main pathological feature is immune cells infiltration, proliferation of fibroblast-like synoviocytes (FLSs) and cartilage erosion [98, 99]. It has been proven that epigenetic regulation is involved in RA pathogenesis [100] and studies of m6A modifications in RA are increasingly significant in recent years.

FLSs are not only the main cells involved in joint destruction of RA, but also responsible for synovial inflammation by releasing inflammatory cytokines like interleukin-6 [101, 102]. Recently, Ye et al. using single-cell analysis identified IGFBP2 and METTL3 were key factors in regulating m6A of NPR3 and GHR in synovial fibroblasts, and then mediated the development and progression of RA [103]. It has been revealed that overexpression of METTL3 not only promoted proliferation, migration and invasion, but also increased the expression of inflammatory cytokines in RA-FLSs via the NF-κB signaling pathway [104]. Wang et al. confirmed that METTL3 overexpression inhibited releasing inflammatory cytokines of macrophages in RA through NF-κB signaling [105]. Furthermore, in Zhang’s study, METTL3 coordinated with YTHDF2 to enhance the inflammatory response in monocytes depend on suppressing the expression of PGC-1α [106]. Together that the immunopathogenesis of RA is complex that METTL3 can play different roles in regulating inflammatory response of different target cells in RA. The reasons may attribute to: Different signaling pathways and different immunocytes. Wang et al. investigated that METTL3 regulated macrophages through NF-κB signaling, meanwhile, Zhang et al. investigated that METTL3 regulated monocytes through suppressing PGC-1α. The roles of METTL3 in inflammatory response of of RA are limited, which may be the future research directions.

Except for METTL3, studies also revealed that METTL14 and ALKBH5 participated in migration, invasion, proliferation and related inflammatory response of RA-FLSs [107, 108]. For example, it has been reported that METTL14 regulates TNFAIP3 expression via m6A-related mRNA stability and involved the inflammatory response of active rheumatoid arthritis [109]. Tan et al. shown that METTL14 improved the mRNA stability of LASP1 through m6A modification and promoted FLSs activation via the LASP1/SRC/AKT axis in RA [107]. Furthermore, Xu et al. reported that ALKBH5 and YTHDF2 regulated m6A modification of MYO1C and contributed to synovial aggression and joint destruction in RA [110]. Mechanistically, ALKBH5 enhances JARID2 mRNA stability through IGF2BP3 and suppressed NLRP3 mRNA expression in cooperation with YTHDF2 are crucial for proliferation, migration, and invasion of RA FLSs [108, 111].

METTL3, METTL14 and ALKBH5 may work as therapeutic target for relieving RA due to crucial regulatory roles in RA progress. For example, Shi et al. found that METTL3 knockdown inhibited inflammatory response in human RA-FLSs and rat AIA-FLSs [104]. METTL14 silencing was proved to relieve RA progression through LASP1/SRC/AKT signaling pathway [107]. And Xiao et al. reported that ALKBH5 inhibited RA progression by suppressing NLRP3 through YTHDC2 [111]. These findings provide novel sights for developing clinical treatment strategies targeting METTL3, METTL14 and ALKBH5. In addition, through systemically analyzing the roles of m6A modifications in RA based on gene expression profiling data, novel targets were identified for RA clinical diagnosis and therapy, such as, Geng et al. found that IGF2BP3 and YTHDC2 could be used to diagnose RA accurately [112]. Besides, WTAP was involved in the m6A modification of ETS1 and regulated the macrophage polarization progression in RA [113]. Furthermore, Song et al. reported that the therapeutic benefits of infliximab can be predicted via the m6A diagnosis model, consist of 20 m6A regulators. This m6A diagnosis model classified RA patients into three clusters with distinct molecular and cellular signatures. And patient in cluster C with adaptive lymphocytes and NK-mediated cytotoxicity signatures was significantly benefited from infliximab therapy [114]. It is more believable that theoretical targets from bioinformatics analysis can be confirmed by experiments like methylated RNA immunoprecipitation, CCK8 assay, RT-qPCR, Western blot and so on. Through cross-validated work based on datasets and experiments, Lin et al. have reported that TGM2 can be a therapeutic target, regulating RA-FLS proliferation and apoptosis via activating NF-κB signaling [115].

### m6A and osteoporosis

OP has become a global health problem mainly among postmenopausal women and elder people, characterized by decreasing bone mineral density, degrading bone microarchitecture and increasing bone fragile [116, 117]. And high incidence of disability and mortality due to osteoporotic fracture can be observed in OP patients. It is observed that the cumulative mortality rate is 69.38% and the 1 year mortality rate increases by 2% per year for patients with osteoporotic hip fracture from 1999 to 2015 [118]. Bone homoeostasis, maintained by osteoblasts, osteoclasts and bone marrow mesenchymal stem cells (BMSCs), is tightly associated with pathogenesis of OP [119]. And recent studies have revealed the molecular mechanisms of m6A modifications on osteoblasts, osteoclasts and BMSCs [120]. We show the functions of m6A regulators in OP in Table 2.

**Table 2:** Functions of m6A regulators in osteoporosis

m6A regulators	Cells	Animals	Expression of m6A regulators	Pathway	Biological function	Reference
METTL3	mouse bone marrow mononuclear macrophages	OVX mouse model	Up	EGR1/METTL3/ CHI3L1	Promoted osteoclast differentiation and osteoporosis development	[121]
	BALB/c mice BMSC	–	Up	MHL/METTL3	Enhanced osteoblastogenesis	[122]
	adipose stem cells in OP rats	OVX mouse model	Down	–	Decreased osteogenic differentiation capacity	[123]
	HUXMA -01 BMSCs	Female C57BL/6 J mice	Up	METTL3/ MIR99AHG/ miR-4660	enhanced the osteogenic differentiation	[124]
	OP mouse BMSCs	OVX mouse model	Down	Wnt signaling pathway	Decreased osteogenic potential	[125]
	Human OP BMSCs	–	Down	MRTTL3/ LINC00657/ miR-144-3p/ BMPR1B	Promoted osteogenic differentiation	[126]
	MC3T3-E1 cells, Balb/c mouse BMSCs	Diabetic bone loss rat model	Up	METTL3/ ASK1-p38	Activated osteoblast ferroptosis	[127]
	Human BMSCs	OVX mouse model	Up	piRNA-36741/ METTL3/ BMP2	Promoted osteogenic differentiation	[128]
	Human BMSCs	OVX mouse model	Down	METTL3/ pre-miR-320/ RUNX2	Inhibited osteogenic differentiation	[129]
	Mouse BMSCs	OVX mouse model	Down	METTL3/ PTH /Pth1r	Impaired bone formation, osteogenic differentiation potential and increased marrow adiposity	[130]
METTL14	Murine RAW264.7 and MC3T3-E1 cell lines	–	Up	METTL14/ NFATc1/ YTHDF1-2	Decreased bone resorption of osteoclasts	[131]
	Human BMSCs	–	Up	METTL14 / miR-873	Inhibited osteogenic proliferation and differentiation	[132]
	Human BMSCs	OVX mouse model	Down	METTL14/ GPX4	Promoted osteoclastogenesis and bone resorption	[133]
	Mouse BMSCs	OVX mouse model	Down	METTL14/ SIRT1	Suppressed osteoblast differentiation and promoted osteoclast differentiation	[134]
	Human BMSCs	OVX mouse model	Down	METTL14/TCF1/ RUNX2	Suppressed osteoblast differentiation and promoted osteoclast differentiation	[135]
	Human BMSCs	OVX mouse model	Down	METTL14/ SMAD1/IGFBP1	Inhibited osteogenic differentiation	[136]
	Human BMSCs	OVX mouse model	Down	METTL14/ IGF2BPs/Beclin-1	Inhibited osteogenic differentiation and promoted osteoclast differentiation	[137]
	Mouse ASCs	OVX mouse model	Down	METTL14/ Notch1	Inhibited osteogenic differentiation	[138]
	Human BMSCs	OVX mouse model	Down	miR-103-3p/ METTL14	Inhibited osteoblast proliferation, differentiation, and matrix mineralization	[139]
WTAP	Human BMSCs	OVX mouse model	Down	WTAP/DGCR8 miR-29b-3p/ HDAC4	Inhibited osteogenic differentiation and promoted adipogenic differentiation	[140]
	Human BMSCs	OVX mouse model	Down	WTAP/YTHDC1/miR-181a and miR-181c/SFRP1	Inhibited osteogenic differentiation and promoted adipogenic differentiation	[141]
FTO	Mouse RAW264.7 cells	diabetic mouse model	Down	FTO/ TLR4	Promoted osteoclast differentiation	[120]
	Human BMSCs	–	Down	FTO/YTHDF1/ PPARG	Inhibited osteogenic differentiation	[142]
	–	OVX mouse model	UP	FTO/ NF-κB/ NFATc1	Promoted bone resorption and osteoclastogenesis	[143]
	Human BMSCs	OVX mouse model	Up	FTO/Runx2	Promotes osteoporosis, inhibited osteogenic differentiation	[144]
	Human BMSCs	OVX mouse model	Up	miR-22-3p/ FTO/MYC/PI3K/AKT	Promotes osteoporosis, inhibited osteogenic differentiation	[145]
	Mouse BMSCs	–	Down	miR-149-3p/FTO	Inhibited the adipogenic differentiation	[146]
ALKBH1	Mouse BMSCs	Alkbh1 knockout mice model	Down	ALKBH1/ optn	Reduced bone mass and increased marrow adiposity	[147]

Osteoclasts are responsible for bone resorption which is important for maintaining bone homoeostasis [119]. Increasing studies have shown that m6A methylation is involved in OP by regulating osteoclast differentiation [121, 133]. Deng and colleagues found that METTL14 was downregulated in postmenopausal osteoporotic women and overexpression of METTL14 can suppress osteoclast formation to ameliorate osteoporosis by stabilizing GPX4 [133]. Moreover, Wang et al. illustrated that METTL14 could alleviate OP via upregulating m6A level of SIRT1 mRNA [134]. Meanwhile, FTO and WTAP participate in alleviating OP through negatively regulation of osteoclast differentiation [120, 140]. Furthermore, Li et al. revealed that METTL3 regulated osteoclast differentiation and function through different mechanisms involving ATP6V0D2 mRNA degradation mediated by YTHDF2 and TRAF6 mRNA nuclear export [148]. And EGR1 positively promotes osteoclastogenesis in osteoporosis by increasing METTL3 and CHI3L1 levels [121]. In addition, YTHDF1 was reported that promoted inflammatory osteoclast differentiation by regulating ER stress and TNFRSF11A mRNA stability [149].

Bone homoeostasis is a dynamic process including removing old bone and promoting new bone formation. Conversed with osteoclasts for bone absorption, m6A contribute to bone homoeostasis by regulating osteoblast activity for new bone formation [150]. Wang et al. found that METTL14 protects against OP via TCF1/RUNX2 axis [135]. Interestingly METTL14, targeted by miR-103-3p, can also inhibit osteoblast activity to promote OP [139]. Likewise, METTL3 was found to promote osteoblast differentiation through piRNA-36741 overexpression [128] and activate the ferroptosis in osteoblasts via ASK1-p38 signaling pathway in diabetic bone loss [127]. Moreover, Zhang et al. reported that METTL3 promoted osteoblast differentiation via Smad signaling and MAPK signaling by stabilizing Smad7 and Smurf1 in YTHDF2-dependent manner [151]. However, METTL3 and YTHDF2 mediated osteoblast apoptosis by regulating endoplasmic reticulum stress during LPS-induced inflammation [152]. Furthermore, FTO was reported that play important function in regulating the maintenance of bone mass and protecting osteoblasts from genotoxic damage [153]. Wu and colleagues demonstrated that YTHDF1 regulated osteogenesis of MC3T3-E1 cells under hypoxia via enhancing the stability of THBS1 [154]. In addition, natural compound Ecliptae herba and wedelolactone can enhance the expression of METTL3 to upregulate the level of HIF-1α, VEGF-A, and RASSF1 and then regulating osteoblastogenesis [122].

**Fig. 2:**
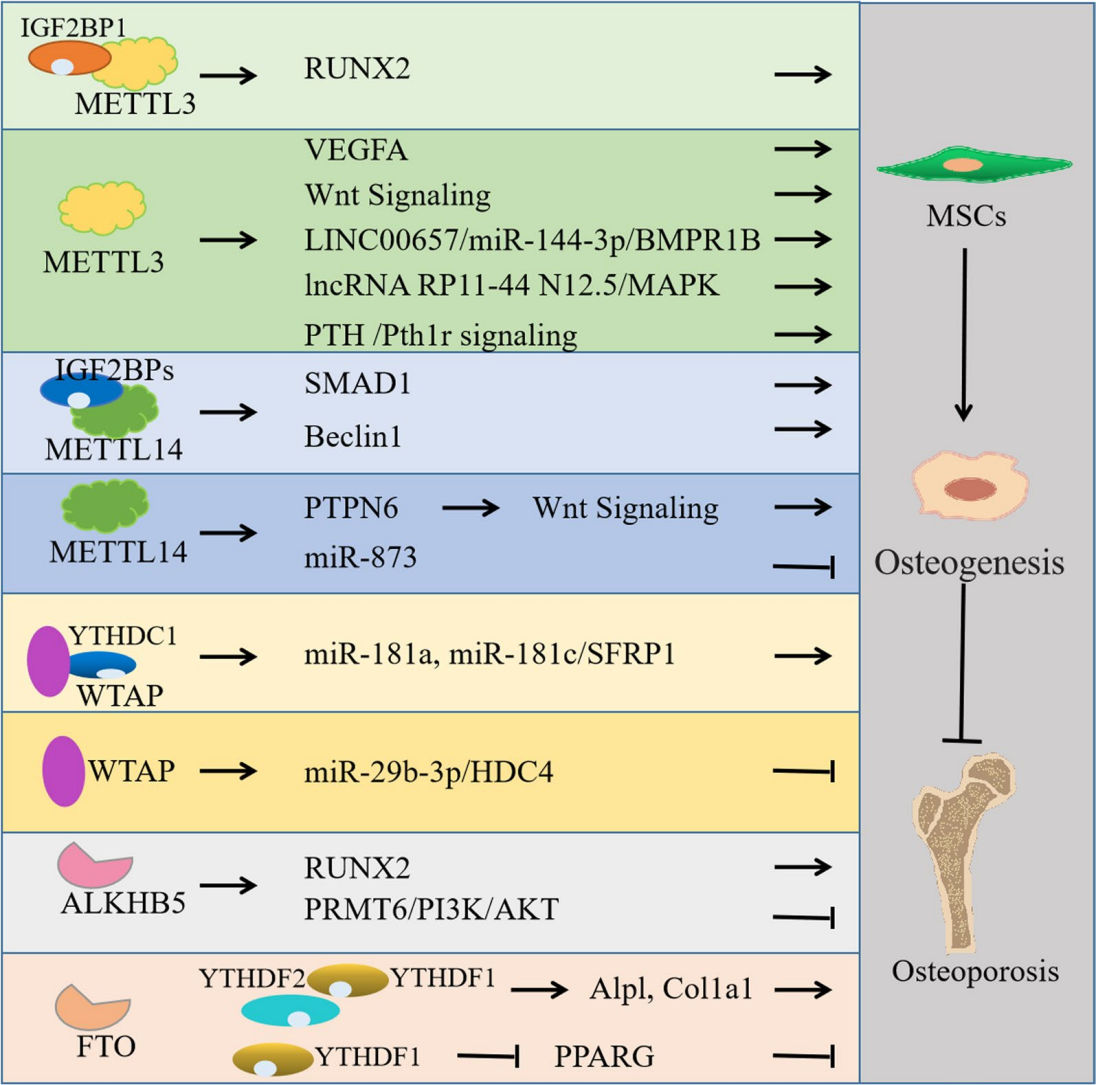
Structural schematic diagram of m6A related protein in regulating osteogenesis in osteoporosis. The role of m6A writers, easers work with readers in regulating the osteogenesis of BMSC and then affacting osteoporosis

As the cellular source for bone reconstruction, BMSCs are known as the ability of self-renewal and multilineage differentiation [119]. And whether BMSCs differentiate into osteogenic cells or adipocytes is linked to the pathogenesis of OP. Recent studies have revealed several mechanisms of m6A modifications to promoted osteogenic differentiation and inhibited adipogenic differentiation of BMSCs [137, 141, 147]. Growing evidences have shown the relationship between m6A modification and BMSC differentiation. We show in Fig. 2 the schematic model of m6A related protein in regulating osteogenesis in osteoporosis. Among these studies, Liu et al. identified that WTAP-mediated m6A methylation regulated BMSCs differentiation through the miR-29b-3p/HDAC4 axis [140]. You and coworkers found that upregulation of WTAP promoted MiR-181a and miR-181c expression via YTHDC1 recognization which sequentially inhibited mRNA expression of SFRP1 to promote BMSCs osteogenic differentiation [141]. Accumulating evidences have reported that METTL3 is another key m6A regulator playing essential roles in BMSCs differentiation. Cooperating with MIR99AHG and LINC00657, METTL3 respectively increases the expression of BMPR1B by sponging miR-144-3p and targets miR-4660 to promote osteogenic differentiation of BMSCs [124, 126]. Moreover, METTL3 overexpression can protect BMSCs from OP via PTH /Pth1r, pre-miR-320/RUNX2 and the Wnt signaling pathway [125, 129, 130]. Furthermore, METTL14, FTO, YTHDF1 and ALKBH1 have been confirmed to regulate BMSCs differentiation, which will help to understand the molecular mechanisms of OP deeply and develop potential therapeutic strategies for OP [132, 142, 147, 155].

Taken together, m6A modifications, including METTL3, METTL14, WTAP, FTO, YTHDF1 and ALKBH1, play essential roles in regulating bone homoeostasis. METTL3 and METTL14 can be novel targets for OP treatment due to diverse regulatory roles on osteoblasts, osteoclasts and BMSCs. For example, METTL14 overexpression suppressed osteoclast differentiation via enhancing GPX4 mRNA stability [133], and METTL14 overexpression increased SIRT1 mRNA expression to promoted osteoblast differentiation [134], and METTL14 overexpression was found to promote the osteogenic differentiation ability of BMSCs [137]. Although diverse regulatory roles of METTL3, METTL14 and FTO show the novel targets for OP treatment, there is lack of research to develop therapeutic strategies targeting METTL3 and METTL14. And m6A regulators identified as therapeutic targets for OP are limited, thus, more attention should be paid to validating the mechanisms of potential therapeutic targets and developing future treatment strategies for OP.

### m6A and osteosarcoma

OS is the most malignant bone tumor mainly occurring in children and adolescents, and the prognosis of OS is poor due to strong aggression, early metastasis, fast growth and high mortality. The definitive diagnosis of OS needs bone biopsy, including needle biopsy and incisional biopsy, and the standard therapeutic strategies consists of neoadjuvant chemotherapy pre/postoperatively and surgical resection [156, 157]. Recently, m6A methylation has become a hot blot in understanding the molecular mechanism of OS pathogenesis to overcome the delayed diagnosis, low survival rate, metastasis and recurrence of OS [158]. We summarize the recent findings related to m6A methylation in OS in Table 3.

**Table 3:** Expression of m6A modulators and their functions in osteosarcoma

m6A regulators	Cells/animals	Samples	Expression of m6A regulators	Targets	Function	Reference
METTL3	MG-63 and U2OS cell lines	Human OS tissue	UP	MALAT1	Promoted proliferation and migration	[159]
	SaOS-2, HOS cell lines	Human OS tissue	UP	CircRNF220	Promoted proliferation and invasion	[160]
	MG-63 and HOS cell lines	Human OS tissue	UP	ZBTB7C	Promoted proliferation and suppressed apoptosis	[161]
	Saos2, SJSA1, MG63, HOS, and U2OS cell lines	Human OS tissue	UP	DANCR	Promoted proliferation, migration and invasion	[162]
	Four-week-old nude mice	Human OS tissue	UP	ATG5	Promoted glycolysis, autophagy and progression	[163]
	SAOS2, MG63, U2OS cell lines	Human OS tissue	UP	COPS5	Promoted migration and invasion	[164]
	MG63, HOS, U2OS, SAOS2 cell lines	–	UP	DIRAS1	Promoted proliferation and invasion, suppressed apoptosis	[165]
	HOS, U2OS, SAOS2 cell lines	Human OS tissue	UP	HDAC5	Promoted proliferation and metastasis	[166]
	MG63, HOS, U2OS, SAOS2 cell lines	Human OS tissue	UP	TRAF6	Promoted metastasis	[167]
	MG63, U2OS cell lines	Human OS tissue	UP	CircNRIP1	Promoted proliferation, migration and suppressed apoptosis	[168]
	MG63, HOS, U2OS, SAOS2 cell lines	Human OS tissue	Down	TRIM7	Promoted migration, invasion and chemoresistance	[169]
	MG63, HOS, U2OS, SAOS2 cell lines	–	UP	ATAD2	Promoted proliferation and invasion	[170]
	MG63, 143B, U2OS, SAOS2 cell lines	Human OS tissue	UP	DRG1	Promoted migration and suppressed apoptosis	[171]
	MG63, HOS, U2OS, SAOS2 cell lines	Human OS tissue	UP	LEF1	Promoted proliferation, migration and invasion	[172]
METTL14	MG63, HOS, U2OS, SJSA-1 cell lines	Human OS tissue	UP	MN1	Tumor progression and chemotherapy resistance	[173]
	MG63, HOS, U2OS, SAOS2 cell lines	–	UP	DIRAS1	Promoted proliferation, invasion and migration	[165]
	MG63, HOS, U2OS, SAOS2 cell lines	Human OS tissue	Down	TRIM7	Promoted migration, invasion and chemoresistance	[169]
	U2OS and 143B cell lines	–	Down	Caspase-3	Promoted proliferation, migration, invasion	[174]
FTO	MG63, HOS, U2OS, SAOS2 cell lines	Human OS tissue	Down	KLF3	Promoted proliferation, invasion, suppressed apoptosis	[175]
	HEK293T, HOS, U2OS, SJSA-1 cell lines	Human OS tissue	Up	DACT1	Promoted proliferation and metastasis	[176]
WTAP	HOS, U2OS cell lines	Human OS tissue	Up	ALB	Promoted migration, invasion and tumor growth	[177]
	SaOS2, U2OS, MG63, HOS, and 143B cell lines	Human OS tissue	Up	SNHG10	Promoted proliferation, migration and suppressed apoptosis	[178]
	MG63, SAOS2 cell lines	Human OS tissue	Up	FOXD2-AS1	Promoted migration, invasion and tumor growth	[179]
	MG63, HOS, U2OS, SAOS2 and 143B cell lines	Human OS tissue	Up	HMBOX1	Promoted proliferation and metastasis	[180]
ALKBH5	KHOS, U2OS cell lines	Human OS tissue	Down	STAT3	Promoted proliferation and tumorigenicity	[181]
	143B, U2OS, and SAOS2 cell lines	–	Down	USP22, RNF40	Promoted proliferation and progression	[182]
	U2OS cell lines	–	Down	YAP/miR-181b-5p	Promoted growth, invasion and suppressed apoptosis	[183]
	LM7, SAOS2, HOS, U2OS, MG63 and 143B cell lines	Human OS tissue	Down	PVT1	Promoted proliferation and tumorigenesis	[184]
RBM15	143B, HOS, MG‐63, U20S SJSA1, SAOS2 cell lines	Human OS tissue	UP	Circ-CTNNB1	Promoted glycolysis and OS progression	[185]
	HOS, MG63, MNNG, and 143B cell lines	–	UP	RRN3, DIDO1	Promoted invasion, migration, and metastasis	[186]
KIAA1429	A673 and SKNMC cell lines	–	UP	STAT3	Promoted proliferation and tumorigenicity in ES	[187]
	MG63 and U2OS cell lines	–	UP	AK2/STAT3	Promoted proliferation, migration, and invasion	[188]
YTHDF1	MG63, U2OS, HOS, SAOS2 cell lines	Human OS tissue	UP	CNOT7	Promoted proliferation, migration and invasion	[189]
	143B cell line	-	DOWN	AC004812.2	Promoted growth, immune cell infiltration	[190]
YTHDF2	MG63, HOS, U2OS, SAOS2 cell lines	Human OS tissue	Down	KLF3	Promoted proliferation and invasion, suppressed apoptosis	[175]
	MG63, HOS, U2OS, SAOS2 cell lines	Human OS tissue	DOWN	TRIM7	Promoted migration and invasion	[169]
	MG63, U2OS, and 143B cell lines	Human OS tissue	DOWN	miR-766	Promoted growth and invasion	[191]
YTHDF3	MG63, SAOS2 cell lines	–	UP	PGK1	Promoted proliferation and aerobic glycolysis	[192]
IGF2BP1	MG-63 and HOS cell lines	–	UP	ERRα	Chemoresistance	[193]
	143B cell line	–	DOWN	AC004812.2	growth, immune cell infiltration↑	[190]
	U2OS, HOS, SAOS2 cell lines	–	UP	MSC-Exo-150	proliferation, migration, invasion↑ apoptosis↓	[194]
	–	Human OS tissue	UP	microRNA-150	Promoted metastasis and recurrence, chemoresistance	[195]
	U2OS, HOS, SAOS2 cell lines	Human OS tissue	Up	IGF2, GLI1, CD44	Promoted cell survival and proliferation↑	[196]
IGF2BP2	MG63, HOS, U2OS, SJSA-1 cell lines	Human OS tissue	UP	MN1	Promoted tumor progression and chemotherapy resistance	[173]
	MG63, U2OS, HOS, 143B, and SAOS2 cell lines	Human OS tissue	UP	HCG11	Promoted cell proliferation, DNA replication↑	[197]
	SAOS2, MG63, HOS and U2OS cell lines	–	UP	DDX11-AS1	Promoted proliferation, metastasis	[198]
ELAVL1	MG63, 143B, U2OS, SAOS2 cell lines	Human OS tissue	UP	DRG1	Promoted proliferation and invasion	[171]

Proliferation, migration, invasion and metastasis are the main biological processes of OS, of which the underlying mechanisms may help to understand the pathogenesis and prognosis of OS [199]. It is reported that METTL3 plays an essential role in promoting OS progression by cooperating with noncoding RNAs, circRNAs and other targets [159–161]. For example, Zhang et al. found that METTL3 promoted the proliferation and migration of OS via increasing the stability of MALAT1 [159]. And inhibiting MALAT1 in a METTL3-dependant manner suppresses cell migration and invasion in Ewing's sarcoma through miR-124-3p/CDK4 axis [200]. Moreover, Zhou et al. found that silencing of another METTL3-mediated noncoding RNA DANCR can also inhibit OS cells proliferation, migration, and invasion [162]. METTL3 and METTL14 co-treatment suppressing DIRAS1 expression can reserve the inhibitory effect on malignant behaviors of HOS cells [165]. Furthermore, evidence have shown that circRNF220 and circNRIP1 are identified as oncogenic roles in OS progression via METTL3 methylation. And METTL3-induced circRNF220 and circNRIP1 promote OS proliferation, invasion by modulating miR-330-5p/survivin axis or sponging miR-199a respectively [160, 168]. In addition, targeting the METTL3/ZBTB7C axis, METTL3/USP13/ATG5 axis, METTL3/COPS5 axis, METTL3/LEF1/ Wnt/β-catenin axis, METTL3/TRAF6 axis and METTL3/HDAC5/miR-142-5p/ARMC8 axis may help to understand OS pathogenesis and develop the novel strategies for OS therapy [161, 163, 164, 166, 167, 172].

Besides METTL3, KIAA1429, YTHDF3, METTL16, WTAP, RBM15 and ALKBH5 are identified to regulate OS progression [15, 181, 185, 187, 192, 201]. It is reported that KIAA1429 knockdown decreases the activity of JAK2/STAT3 signal to decreased cell proliferation, migration, and invasion of OS, which can be rescued by JAK2/STAT3 stimulator colivelin [188]. And KIAA1429 can also act as a crucial gene to regulate Ewing sarcoma progression after CRISPR-Cas9 knockout [187]. Moreover, researchers found that aerobic glycolysis is essential to make sure OS cells obtain metabolic survival advantage compared with other cells [192]. Yang et al. have proved that circ-CTNNB1 interacted with RBM15 drives aerobic glycolysis and OS progression by elevating key aerobic glycolysis genes expression, such as PGK1 [185].

Increasing evidences have reported that m6A regulators can act as prognostic biomarkers of OS [202]. For example, high expression of METTL3, IGF2BP2, YTHDC1, KIAA1429 and HNRNPA2B1 and low expression of FTO, METTL14 and YTHDF2 have been identified to result in poor prognosis of OS [4, 203, 204]. Through a comprehensive bioinformatic analysis, Kaplan–Meier and Cox regression analyses, Li et al. concluded that low expression of FTO was prognostic biomarker for poor prognosis in OS [203]. Interestingly, upregulated FTO was proved to predict a poorer prognosis of OS via the FTO/DACT1 axis [176]and overexpression of FTO was correlated with low prognosis survival of OS patients by regulating KLF3 expression [205]. In summary, the findings between bioinformatic analysis and experimental validation may be different. Thus, more experiments are necessary to validate the prognostic biomarkers identified through comprehensive bioinformatic analysis.

Effective neoadjuvant chemotherapy is helpful to improve survival of OS, but chemotherapy resistance is a big challenge for OS therapy [206]. Thus, it is necessary to explore the mechanism of chemotherapy resistance. Recently, researchers have observed higher levels of METTL3, ALKBH5, METTL14 and IGF2BP1 in chemotherapy-resistant OS cells [173, 193, 207], and METTL14 and YTHDF2 are associated with multiple drug sensitivity in Ewing's sarcoma. Different from higher levels of METTL3 observed in Wang’s study [207], Zhou et al. found that lower levels of METTL3 and YTHDF2 contributed to higher TRIM7 expression which promoted chemotherapy resistance in OS through ubiquitination of BRMS1 [169]. The possible reasons of this phenomenon include but not limited to: (1) experiment methods and materials between Wang’s study and Zhou’s study are different; (2) different RNA binding proteins can lead to different functional effects of m6A methylation on downstream processes, like translation [208]. Furthermore, IGF2BP1/ERRα axis can regulate the metabolic reprogramming of Doxorubicin-resistant OS cells [193] and METTL14-IGF2BP2-MN1 panel is responsible for all-trans-retinoic acid resistance in osteosarcoma [173]. The above findings suggest that m6A modification can provide a novel sight in understanding mechanism of chemotherapy resistance in OS and develop effective chemotherapy strategies for OS. Nevertheless, more studies are still needed to enrich our limited understanding of the relationship between m6A methylation and chemotherapy resistance in OS. And possible controversial findings of chemotherapy resistance in OS should be validated by further detailed studies.

## Discussion

### Clinical value of m6A modifications for SSD

Given that dysregulation of m6A modifications plays a crucial role in: (1) Affecting ECM degradation, immune microenvironment and apoptosis in OA chondrocyte; (2) Regulating inflammatory response of immunocytes, and rheumatoid fibroblast-like synoviocytes proliferation, migration and invasion; (3) Maintaining bone homoeostasis of OP, including osteoblasts, osteoclasts and BMSCs; (4) Proliferation, and metastasis of OS [108, 209]. It is believable that m6A modifications can offer novel ideas for early diagnosis, clinical therapy and prognosis of skeletal system diseases [210]. For example, Luo et al. firstly revealed that the expression of ALKBH5, FTO, and YTHDF2 in peripheral blood of RA patients were significant low compared to control patients [211]. YTHDF2 was significant decreased while IL-1β expression was increased in RA patients’ peripheral blood mononuclear cells [212]. These findings make it possible to early diagnose RA, even skeletal system diseases, via detecting the level of m6A regulators in peripheral blood. Moreover, Bian and colleagues concluded that YTHDF2 was a crucial m6A regulators with high diagnostic value in OA, based on the protein and mRNA contents of YTHDF2 were significantly lower in OA patients via WB and qRT-PCR, which was consistent with the results of bioinformatic analysis [213]. Similarly, in Qiao’s study, METTL16, CBLL1, FTO, and YTHDF2 were applied to build a diagnostic model in OP. METTL16 and FTO were identified as risk factors in promoting OP progress, whereas CBLL1 and YTHDF2 were protective factors [214]. Furthermore, m6A modified noncoding RNAs were also could be act as biomarkers for disease diagnosis. Such as, Chen et al. reveled that hyper-methylated hsa_circ_0007259 activated STAT3 signaling pathway via sponge miR-21-5p and could be acted as a potential biomarker in RA [215]. Besides, it has been reported that m6A-related lncRNAs regulated the tumor immune microenvironment and predicted the overall survival of OS patients [190, 216]. Although researchers have made progress in exploring the diagnosis value of m6A methylation in SSD, further detailed studies are needed to verify the effectiveness, accuracy and feasibility of these findings in larger samples.

In addition to diagnosis, researchers have explored treating SSD via targeting m6A regulators. For example, Chen et al. found that knockdown of METTL3 could inhibit SASP expression in OA-FLS and relieve the cartilage destruction in DMM mice model [78]. In Ye’s study, overexpression of ALKBH5 could alleviated MSC senescence through inhibiting the expression of CYP1B1 and alleviating mitochondrial dysfunction [85]. Moreover, Wang and his coworkers reported that knockdown METTL3 could inhibit EGR1 expression to suppress osteoclastogenesis, and then alleviate OP [121]. These results illustrated that targeting m6A regulators expression might be novel strategy for SSD treatment. Besides modified the expression of m6A regulators in the molecular level for SSD therapy, natural compounds or small molecules were also shown significant role in regulating the expression of m6A regulators. Such as, Natural Compound Radicicol was identified as a potent FTO inhibitor and exhibited a dose-dependent inhibition of FTO demethylation activity with an IC50 value of 16.04 μM [217]. Moreover, Gao et al. have shown that acetaminophen treatment could recover m6A levels and related protein expression mainly including, downregulating METTL3 and upregulating ALKBH5, and suppress inflammatory cytokines secretion in IL-1β-treated chondrocyte cells [218]. Moreover, Chinese Ecliptae herba extract and its component wedelolactone enhances osteoblastogenesis of BMSCs by targeting METTL3-mediated m6A methylation of HIF-1α, VEGF-A, and RASSF1 [122].

And recent studied found that drug resistance of OS may attribute to m6A methylation. Evidence suggested that IGF2BP1/ERRα axis was involved in metabolic reprogramming which led to chemoresistance of OS cells [193]. What’s more, METTL3 and YTHDF2 METTL3 and YTHDF2 mediated N6-Methyladenosine modification of TRIM7 positively regulates chemoresistance in OS through ubiquitination of BRMS1 [169]. However, the understanding of m6A methylation in clinical application of SSD is still in infant stage, lacking exploring signaling pathways, m6A methylation targeted drugs and developing effective therapeutic strategies. And more studies should focus on explore the mechanisms of m6A methylation in diagnosis, clinical therapy and prognosis of SSD based on the breakthroughs of m6A methylation in SSD pathogenesis.

### Challenges of m6A methylation in SSD

First, recent advances of m6A methylation in SSD mainly focus on METTL3, METTL14, WTAP, FTO, ALKBH5, other m6A writers (eg. METTL16, RBM15/15B and KIAA1429) and m6A readers (eg. YTHDF1/2/3, YTHDC1/2, HNRNPC/G/A2B1 and IGF2BP1/2/3) should obtain more research attention. Second, study of m6A methylation in immune microenvironment of SSD is limited. It is known that m6A interacted with noncoding RNA is associated with biological processes of many diseases. Thus, the role of m6A and noncoding RNA in immune microenvironment of SSD might be a hot blot for future research. Third, the dual roles of m6A methylation in SSD may make it more complex to understand SSD pathogenesis. For example, METTL3 was proved to promote osteoclastogenesis in osteoporosis [121], meanwhile, Tian et al. showed that upregulation of METTL3 could enhance osteoblastogenesis of BMSC [122]. The reasons of the dual roles of METTL3 and METTL14 in OP may attribute to: (1) Different study purposes. Tian et al. explored the molecular mechanism of Ecliptae herba on osteoblast differentiation in OP while Wang et al. investigated EGR1-mediated METTL3/m6A/CHI3L1 axis in OP. (2) Different research material and methods. Tian et al. treated BMSC isolated from BALB/c mice with MHL while Wang et al. used OVX mouse osteoporosis model and stimulated mouse BMMs with cytokines. (3) Different signaling pathways. Tian et al. found that MHL targeted METTL3 and then uoregulated METTL3 to promote osteoblastogenesis. And Wang et al. found that EGR1 upregulated METTL3 and then increased CHI3L1 level to promote osteoclastogenesis. Although the dual roles of m6A regulators are limited in current studies, further studies are needed to reveal the underlying mechanism of m6A methylation dual roles.

## Conclusion

Taken together, this article summarizes the recent advances of regulatory role of m6A modifications in common SSD (OA, RA, OP and OS). Although m6A modifications have brought significant breakthroughs for underlying mechanisms of biological and pathological processes in SSD, there is a lack of detailed studies on m6A modification involved in clinical diagnosis, treatment and prognosis of SSD. Hopefully, future studies will show deeper understand of m6A regulation in SSD pathogenesis and make the clinical value of m6A modification come true.

## Data Availability

Not applicable.

## Abbreviations

OS: Osteosarcoma
SSD: Skeletal system disease
m6A: N (6)-methyladenine
OA: Osteoarthritis
OP: Osteoporosis
RA: Rheumatoid arthritis
mRNA: Messenger RNA
lncRNA: Long non-coding RNA
YTH: RNA binding protein DC1/2
MDR1: Multidrug resistance protein 1
ALB: Albumin
RBPs: RNA binding proteins
ELAVL1: ELAV-like protein 1
MTC: Methyltransferase complex;
hm6A: N6-hydroxymethyladenosine
f6A: N6-formyladenosine
ACL-T: Anterior cruciate ligament transection
MIA: Maternal immune activation
BMSC-Exos: Bone marrow stem cell exosome
AIA-FLS: Adjuvant-induced arthritis fibroblast-like synoviocyte
OVX: Ovariectomized
ASC: Adipose stem cell
ECM: Extracellular matrix
LPS: Lipopolysaccharide
DMM: Destabilizing the medial meniscus
FLS: Fibroblast-like synoviocyte
mTOR: Rapamycin
MSC: Mesenchymal stem cell
CCK8: Cell counting kit 8
BMSCs: Bone marrow mesenchymal stem cells
ER: Endoplasmic reticulum
circRNAs: Circular RNAs
ES: Ewing’s sarcoma
IL-1β: Interleukin-1β
SASP: senescence-associate secretory phenotype
